# The Impact of COVID-19 on Patient Presentations to the Emergency Department

**DOI:** 10.7759/cureus.26100

**Published:** 2022-06-20

**Authors:** Noumi Chowdhury, Kianna Eurick-Bering, Mariam Hjaige, Ryan Kenerson, Taylor A Revere, Ryan J Reece

**Affiliations:** 1 Department of Emergency Medicine, Michigan State University College of Human Medicine, Flint, USA; 2 Department of Emergency Medicine, University of Michigan, Ann Arbor, USA

**Keywords:** michigan, emergency department, operations, emergency medicine, covid-19

## Abstract

Introduction

As the coronavirus disease 2019 (COVID-19) pandemic continues, it may be useful to elucidate its impact on services in the emergency department (ED). This research project aims to identify and analyze changes in patient presentations and disease severity within the ED at Hurley Medical Center (HMC) in Flint, Michigan, due to the COVID-19 pandemic.

Methods

The study is a retrospective chart review focusing on adults 18 years and above who presented to HMC’s ED. The data collected for the study was obtained from patient charts from February 1, 2019, to July 31, 2019, and from February 1, 2020, to July 31, 2020. Data from the years 2019 and 2020 were analyzed using a combination of independent t-test, chi-square analysis, and regression modeling.

Results

There were a total of 59,345 visits analyzed within the study; 33,648 ED visits within the study were in 2019 compared to 25,697 visits in 2020. There was a significant difference in patient sex between 2019 and 2020 with a larger percentage of males presenting in 2020 versus 2019 (p<0.001). Dispositions also significantly differed in 2020 compared to 2019 with more patients being admitted or dying in the ED (p<0.001). Patients who presented to the ED often presented with more severe illness in 2020 as reflected in increased length of stay in 2020 (p=0.01) and increased case mix index (p<0.001).

Conclusion

The COVID-19 pandemic significantly reduced the total number of ED visits to HMC in Flint, Michigan, in 2020 than in 2019. Notably, patients were more likely to have a longer length of stay, present with more severe illness, and be admitted or die in the ED when compared to 2019. The results from this study can be used for future planning for the next public health emergency.

## Introduction

Background

This article was previously presented as a meeting abstract at the 2021 ACEP Annual Scientific Meeting Research Forum on October 25-28, 2021.

The World Health Organization marked March 11, 2020, as the beginning of the COVID-19 pandemic [1]. The state of Michigan declared a state of emergency in response to COVID-19 on March 10, 2020, and enacted a lockdown order on March 23, 2020, via an executive order from the Michigan Governor’s office [2]. As the pandemic continues, there is a need to highlight the impact of COVID-19 on patients accessing services in the emergency department (ED). Current investigations highlight trends in volume, diagnoses, and disease severity. Ultimately, the current data demonstrate how COVID-19 has affected which patients are presenting and when they are presenting.

The National Syndromic Surveillance Program (NSSP) has been collecting real-time data regarding emergency department visits in the United States during the COVID-19 pandemic and comparing the data to the same time period in 2019. The NSSP data suggests that the number of emergency department visits declined by 42% during the early months of the COVID-19 pandemic; visits decreased from 2.1 million per week in March and April 2019 to 1.2 million per week in March and April 2020 [3]. Not only did the volume of emergency department visits change, but the conditions for which patients sought treatment also changed. Eight of the most common diagnostic categories saw an increase in visits, including diagnoses related to exposure and screening for infectious disease, pneumonia, respiratory failure, and cardiac arrest [3]. Similarly, emergency department services for opioid overdoses have also increased in regional studies [4]. Other diagnostic categories, however, saw declines in visits. In particular, visits for abdominal pain, musculoskeletal pain, essential hypertension, and nonspecific chest pain decreased [3]. Most concerning, however, is the steep decline in visits for acute, life-threatening conditions. NSSP data indicates a 23% decline in visits for myocardial infarction, a 20% decline for cerebrovascular accidents, and a 10% decline for a hyperglycemic crisis [5].

Importance

While it is clear that COVID-19 has altered the number of patients utilizing the ED and impacted what complaints people are willing to come to the ED for, data also suggests that COVID-19 has impacted the severity of presentations. In a multicenter study across five healthcare systems in five states, increases in hospital admission rates in the ED ranged from 22% to 149%, suggesting that patients were presenting with more severe concerns [6]. While limited studies in the United States suggest that ED visits have increased in severity, other studies conducted outside of the United States also found similar results. In one international study, individuals with scores of “1” or “2” on the Canadian Triage and Acuity Scale corresponding to threat to immediate life/limb requiring resuscitation and those potentially requiring resuscitation, respectively, saw the steepest decline in ED visits in comparison to less severe triage scores [7]. The correlation between increased disease severity upon presentation and COVID-19 is not entirely clear as other international studies found no significant difference in triage levels between pre-COVID-19 and COVID-19 [8].

Goals of this investigation

Despite the robust data collection by NSSP, several gaps remain in the knowledge regarding COVID-19 and emergency department changes. First, the current data is largely national and regional. There are no current studies detailing the effects of the COVID-19 pandemic on emergency department visits in Michigan or communities in Michigan such as Flint. Furthermore, although data elucidating the changes in severity for emergency department visits suggests an increase in severity, some studies internationally suggest that there has been no increase in severity, perhaps indicating that severity changes are more specific to geographical locations.

## Materials and methods

Study design

This study was reviewed by the institutional review board (IRB) and was found to be an exempt study. It was a retrospective chart review study investigating emergency department patient visits. The data collected for the study was obtained from existing information recorded in medical charts from February 1, 2019, to July 31, 2019, and from February 1, 2020, to July 31, 2020. Data were aggregated based on month, and each month’s data in 2020 were compared to its respective month in 2019. These six months in 2020 were chosen as this was a six-month snapshot of ED visits that reflect the impact of the pandemic at various times of viral impact in the community.

Setting

This work was conducted at Hurley Medical Center (HMC) in Flint, Michigan, a Level I Trauma Center, Level II Pediatric Trauma Center, and regional burn center that services the Genesee, Lapeer, and Shiawassee counties in Michigan. From the United States Census data, we found that Flint has a larger minority population, lower average property values, lower overall education rate, and higher poverty and unemployment rates than the national average [9].

Selection of participants

The study population included adults 18 years and older. We obtained data via the health information technology (HIT) department’s summary of patient medical records from emergency visits during the time period studied.

Analysis

We aggregated yearly data on demographic variables in terms of sex and race, severity markers such as length of stay hours, and case mix index from the EPIC electronic medical record system. Diagnoses through the categorization of ICD-10 codes were also collected. Each of these elements was compared between data collected in February 2019-July 2019 and February 2020-July 2020. Data were analyzed using an independent t-test and chi-square, as appropriate, using SPSS version 27.0 (IBM Corp., Armonk, NY, USA).

## Results

There were a total of 59,345 visits analyzed within the study, of which 33,648 ED visits occurred in 2019 compared to 25,697 visits in 2020 (Table 1).

**Table 1:** Comparison of Emergency Department Values in 2019 Versus 2020

Year	Number of ED visits
2019	33,648 (56.7%)
2020	25,697 (43.3%)
Study total	59,345

There was a significant difference in patient sex between 2019 and 2020 with an increase in males in 2020 at 47% (n=12,067) in comparison to 2019 at 44.2% (n=15,025). However, there was a decrease in the presentation of females in 2020 at 53% (n=13,630) in comparison to 2019 at 55.8% (n=18,791). There was no statistical difference in the presentation of racial demographics between 2019 and 2020 (Table 2).

**Table 2:** Comparison of Patient Demographics in 2019 Versus 2020

Year	2019 (n=33,648)		2020 (n=25,697)		p-value
	Number	(%)	Number	(%)	
Sex					
Male	14,857	44.2	12,067	47	<0.001
Female	18,791	55.8	13,630	53	
Race					
White	15,025	44.7	11,559	45	0.071
Black	17,175	51	12,899	50.2	
Hispanic	752	2.2	660	2.6	
American Indian and Alaskan Native	114	0.3	90	0.4	
Native Hawaiian and Other Pacific Islander	8	0.02	9	0.035	
Asian	46	0.1	33	0.1	
Other	363	1.1	298	1.2	
Unknown	165	0.5	149	0.6	

The length of stay and case mix index were used as proxy indicators of severity, which displayed a statistically significant increase in 2020. The average length of stay was measured at 6.97 hours in 2020 in comparison to 6.81 hours in 2019. Additionally, the case mix index was 1.93 in 2020 in comparison to 1.65 in 2019 (Table 3). There were also statistically significant changes in disposition with an increase in patient admissions, discharges, and deaths but a decrease in disposition to skilled nursing facilities (Table 4).

**Table 3:** Comparison of Length of Stay and Case Mix Index in 2019 Versus 2020

Year	2019	2020	p-value
Length of stay (hours)	6.81	6.97	0.01
Case mix index	1.65	1.93	<0.001

**Table 4:** Comparison of Disposition in 2019 Versus 2020

Year	2019		2020		p-value
Disposition	Number	(%)	Number	(%)	
Discharge	23,140	68.8	17,721	69	<0.001
Admit	7,116	21.1	5,870	22.8	
Skilled nursing facility	884	2.6	535	2.1	
Death	230	0.7	255	1	

Multiple ICD-10 diagnoses differed between 2019 and 2020. There was an increase in the percentage of infectious diseases, COVID-19, and pneumonia. Respiratory failure/insufficiency/arrest increased in comparison to 2019 (4% (n=1,035) in 2020 versus 2.9% (n=989) in 2019). There was also an increase in nausea/vomiting and generalized signs and symptoms at 8.2% (n=2,107) and 3.7% (n=942) in comparison to 2019 at 6.9% (n=2,320) and 3.2% (n=1,089).

In addition, there was an increase in patients with socioeconomic factors and mental health diagnoses. Socioeconomic factors increased to 1.8% (n=437) in 2020 from 1.6% (n=542) in 2019, and mental health increased to 4.5% (n=1,161) from 4.1% (n=1,376).

An increase in patients with a history or family history of chronic disease and disorders of lipid metabolism was also seen in 2020. In addition, 24.8% (6,373) of the patients with a personal or family history of chronic disease and 6.5% (n=1,660) of patients with disorder of lipid metabolism presented to the ED in 2020 in comparison to 22.9% (n=7,691) and 5.8% (n=1,941) in 2019 (Table 5).

**Table 5:** Comparison of Diagnoses in 2019 Versus 2020

Year	2019		2020		p-value
Diagnoses	Number	(%)	Number	(%)	
Infectious disease	2,682	8	2,181	11	<0.001
COVID-19	0	0	462	1.8	<0.001
General signs/symptoms	1,089	3.2	942	3.7	<0.01
Pneumonia	532	1.6	747	2.9	<0.001
Respiratory failure/insufficiency/arrest	989	2.9	1,035	4	<0.001
Cardiac arrest	166	0.5	125	0.5	0.905
Socioeconomic factors	542	1.6	437	1.8	<0.05
Mental health	1,376	4.1	1,161	4.5	<0.05
Abdominal pain	4,162	12.4	3,210	12.5	0.654
Essential hypertension	7,733	23	5,918	23	0.891
Nausea or vomiting	2,320	6.9	2,107	8.2	<0.001
Sprain	789	2.3	452	1.8	<0.001
Personal/family history of disease	7,691	22.9	6,373	24.8	<0.001
Urinary tract infection	1,491	4.4	995	3.9	<0.01
Sexually transmitted infection	195	0.6	114	0.4	<0.05
Asthma	1,542	4.6	1,158	4.5	0.658
Disorders of lipid metabolism	1,941	5.8	1,660	6.5	<0.001
COPD or bronchiectasis	2,494	7.4	1,823	7.1	0.14
Myocardial infarction	457	1.4	414	1.6	<0.05
Cerebral infarction	175	0.5	153	0.6	0.22

## Discussion

There was a significant increase in indicators of severity such as emergency department length of stay, overall admissions to the hospital, and case mix index (CMI) in 2020 when compared to 2019. There was a statistically significant difference in patient presentation in 2020 by sex, but not by race. In regard to patient disposition, there was a statistically significant increase in the percentage of hospital admissions, discharges, and deaths in 2020 compared to 2019. There was a statistically significant decrease in the number of patients discharged to skilled nursing facilities in 2020 compared to 2019.

The diagnoses for which individuals visited the emergency department also varied between 2019 and 2020. There was a statistically significant increase in the percentage of infectious disease, COVID-19, generalized symptoms, pneumonia, respiratory failure/insufficiency/arrest, patients with socioeconomic factors, mental health, nausea/vomiting, and myocardial infarction presentations in 2020 compared to 2019.

Despite increases in the percentage of the diagnoses listed above, there was an overall decrease in the number of patients presenting with the above diagnoses except for COVID-19, pneumonia, and respiratory failure/insufficiency/arrest. There was a statistically significant decrease in the percentage of sprain, unspecified injury, urinary tract infection, and sexually transmitted infection presentations in 2020 compared to 2019.

This study is the first of its kind to study the impact of COVID-19 on patient presentations to the emergency department in our region in Michigan. The information gathered from this study can be used to prepare the emergency department and downstream hospital operations for subsequent public health emergencies.

Our data further suggest that there was no statistically significant decline in time-sensitive emergencies for myocardial infarction, cerebral infarction, or cardiac arrest despite the constraints of COVID-19. Further work should be directed to investigate the interplay between the social determinants of health in Flint, Michigan, and the safety net in place at HMC in the context of COVID-19.

Limitations

Due to the large sample size of the study, it is difficult to visualize substantial changes in patient presentations using percentages alone. When analyzing large numerical populations, it is observed that small percentage changes represent hundreds of patients. Variable percentages may also be attributed to the decrease in patient presentations in 2020 due to COVID-19.

Our study compared data from February 1, 2019, to July 31, 2019, and February 1, 2020, to July 31, 2020. Therefore, the conclusions and discussion made reflect only part of the ongoing COVID-19 pandemic. Additionally, this study evaluated emergency department hospital visits at a single hospital in Flint, Michigan. Thus, this data is specific to the local population and may not indicate what other hospitals or geographic areas are experiencing in Michigan or the United States. Lastly, one investigative measure, severity, may not be directly comparable to other studies looking at ED visit severity as there are other markers to assess for severity.

## Conclusions

During the first major wave of COVID-19 at Hurley Medical Center in Flint, Michigan, emergency department visits dropped approximately 13% compared to the previous year. However, patients who did present to the emergency department were found to be sicker and tended to get admitted to the hospital. Unlike previous research on this topic, the rate of time-sensitive emergencies (myocardial infarction, stroke, and cardiac arrest) remained flat despite COVID-19 constraints.
